# Plastic Metallic Fillings

**Published:** 1862-10

**Authors:** 


### PLASTIC METALLIC FILLINGS.

*A substitute for the amalgams, metallic oxids, and base metals for filling teeth.*

This material, prepared by Dr. B. Wood, as a cheap filling for teeth, is quite an advance on former attainments. It is called Cadmium Alloy, and is designed to take the place of amalgams, tin, metallic oxids, etc., for filling. In some respects, it is better than any of these ; it is better than amalgam, because it does not contain mercury, and hence an absence of the difficulties that in such a variety of ways occur, or are liable to occur, with that agent. It is better than tin, from the fact that it forms a solid mass ; it is also harder, and is more easily introduced. Some skill and experience in manipulation are necessary, to secure the best results, but when these are attained, a filling can be made with great facility. Fillings can be made with it in cases where tin foil can not be used at all. It can be used in any case where amalgam can.

It is rendered plastic by heat, 140° being sufficient for that purpose ; and hence it may be introduced without giving pain, even in sensitive teeth. In case it should be desirable to remove it, it can be done as easily as it was introduced. It is not as easily acted on by oxygen or chlorine as tin.

Dr. W. furnishes directions to all who use it. It is patented, but no license is required by those who use it. See card in the advertising sheet of this number. T.

